# Jaundice on Presentation Is Associated with Higher In-Patient Mortality and Complications in Patients Admitted for Acute Pancreatitis: A Retrospective Study Based on National Inpatient Sample Database

**DOI:** 10.1155/2022/5048061

**Published:** 2022-10-18

**Authors:** Nishit Patel, Krishna Bodrya, Kirten Patel, Nishi Patel, Dhruvanshu Patel, Ronak Modi, Ayaz Matin

**Affiliations:** ^1^Internal Medicine Department, St. Luke's University Hospital Health Network Bethlehem, PA, USA; ^2^Trumbull Regional Medical Center, Niles, OH, USA; ^3^St. Mary Mercy Hospital, Langhorne, PA, USA; ^4^University of South Columbia (Pharmacy), Columbia, SC, USA; ^5^Gastroenterology Department, St. Luke's University Hospital Health Network, Bethlehem, PA, USA

## Abstract

Pancreatitis usually presents with characteristic abdominal pain, radiological findings, and elevated lipase. The presence of jaundice may hint at a biliary etiology; however, it is not always present. We hypothesized that the presence of jaundice is associated with worse outcomes in patients admitted with pancreatitis. We conducted a retrospective analysis using the National Inpatient Sample, inquiring about patients admitted with pancreatitis with and without jaundice between October 2015 and December 2017. The primary outcome was in-hospital mortality in patients admitted for pancreatitis with and without jaundice. Secondary outcomes were the median length of stay, hospitalization cost, the incidence of ventilator-dependent respiratory failure (VDRF), acute respiratory distress syndrome (ARDS), sepsis, septic shock, dehydration and electrolyte disturbances, and ascites. A total of 1,267,744 patients were admitted with pancreatitis from October 2015 to December 2017. Among them, 8855 (0.7%) had concomitant jaundice on presentation. In-hospital mortality in this group was 4.3%. The patients with pancreatitis and jaundice had higher odds of in-hospital mortality (adjusted odds ratio [aOR]: 1.51, 99% CI 1.35–1.68, *p* < 0.0001) as compared to those without jaundice. Patients with jaundice showed a significantly higher incidence of sepsis (15.2% vs. 9.6%, *p* < 0.0001), septic shock (4.1% vs. 2.9%, *p* < 0.0001), ascites (6.5% vs. 3.1%, *p* < 0.0001), and dehydration and electrolyte disorders (47.6% vs. 43.8%, *p* < 0.0001). Patients with jaundice also had higher total hospital costs ($11,412 vs. $7893, *p* < 0.0001). There was no statistical difference in ARDS, VDRF, and median length of stay. In conclusion, patients admitted for pancreatitis with jaundice had worse outcomes, including in-hospital mortality and complications, compared to those without jaundice.

## 1. Introduction

Acute pancreatitis is a common gastrointestinal presentation in hospitals worldwide, with 70 cases per 100,000 people [1]. It involves inflammation of the pancreas, which can lead to local and systemic complications. Diagnosis usually requires the presence of two out of three of the following: (1) typical abdominal pain, (2) radiographic evidence of pancreatic inflammation, and (3) elevated serum lipase. Common causes of acute pancreatitis include gallstones and alcohol, followed by hypertriglyceridemia, drug-induced, auto-immune, trauma, malignancy, and idiopathic. The presentation of pancreatitis is indistinguishable based on the etiology. Gallstone disease is responsible for up to 70% of acute pancreatitis, and the proposed pathology includes the restricted outflow of pancreatic secretions and occasional bile reflux into the pancreatic duct, leading to pancreatic inflammation [2–6].

Jaundice is defined as yellowish or greenish pigmentation of the skin and sclera due to high bilirubin levels that hint at the presence of underlying diseases involving abnormal heme metabolism, liver dysfunction, or biliary tract obstruction. When seen in conjunction with pancreatitis, jaundice suggests a biliary cause, which could include biliary sludge, anatomical defect of the biliary tract, or impacted gallstones. Demographically, gallstone pancreatitis is more common in females and presents later than hypertriglyceridemia-induced pancreatitis [7–11]. Autoimmune pancreatitis and chronic pancreatitis can lead to biliary strictures resulting in jaundice. Pancreatic cancer (PA) has to be considered a possible etiology in the appropriate clinical setting. Jaundice has been better studied with chronic pancreatitis and PA [12, 13], but data on the co-relation of jaundice and acute pancreatitis are unclear. Shaka et al. derived that biliary pancreatitis was associated with worse outcomes than hypertriglyceridemia-induced pancreatitis [14]. It was associated with worse mortality and complications, including sepsis, septic shock, ascites, non-ST elevation myocardial infarction, and renal failure [14]. Outcomes and prognosis of pancreatitis are determined by two factors that reflect the disease's severity: organ failure and pancreatic necrosis. Necrosis can be evaluated by radiography, and organ failure is defined as: (1) shock, (2) respiratory failure, (3) renal failure, and (4) gastrointestinal bleeding as per Atlanta Symposium (1992). The severity indices used for pancreatitis to predict outcomes do not include jaundice, as jaundice in patients admitted for pancreatitis and its association with outcomes have not been extensively studied. We conducted this study to determine if jaundice is associated with worse outcomes in patients admitted for pancreatitis, irrespective of the underlying etiology. To the best of our knowledge, this study represents the first large-scale nationwide study evaluating outcomes of pancreatitis with jaundice compared to those without jaundice.

## 2. Method

### 2.1. Study Design

We conducted a retrospective cohort study that included adult admission for pancreatitis in the United States from October 2015 to December 2017. The database used was the National (Nationwide) Inpatient Sample (NIS), which covers over 97% of the population across 47 states in the Unite States and the District of Columbia and has been shown to provide reliable inpatient estimates of comorbidities and disease prevalence [15, 16]. International Classification of Diseases (Tenth Revision) (ICD-10) and Clinical Modification Procedure Coding System (CM/PCS) were used to conduct the study. Principal diagnosis represents the admission diagnosis, which for our study is pancreatitis, while secondary diagnosis refers to other assigned diagnoses.

### 2.2. Study Population

NIS data were gathered from October 2015 to December 2017 who had a principal diagnosis of acute pancreatitis (K85). Exclusion criteria were age less than 18 years. The NIS database does not contain any patient identifiers. However, to comply with the research regulations of the home institute of the primary investigator, IRB approval was obtained from the IRB committee at St. Luke's University Health Network in Bethlehem, PA, USA.

### 2.3. Study Outcomes

The primary outcome was to compare inpatient mortality among patients admitted for pancreatitis with and without jaundice. Secondary outcomes in this population include the development of sepsis, septic shock, ascites, dehydration, and electrolyte imbalance. We also compared the median length of hospital stay and total hospitalization charges between the two groups.

### 2.4. Statistical Analysis

Data were analysed using the statistical software SAS 9.4. All data analyses performed utilized the Healthcare Cost and Utilization Project (HCUP) regulations. Chi-squared testing was performed to compare the characteristics between patients with and without jaundice. Multivariate regression analysis was then used to remove the effect of possible confounders and to calculate outcomes. The univariate screening was utilized to confirm the factors that affected the results.

## 3. Results

### 3.1. Patient Characteristics

The NIS database from October 2015 to December 2017 recorded over 71 million hospital discharges, of which 1,267,744 have a principal discharge diagnosis of acute pancreatitis [16]. Of these patients, 0.7% (8855) were classified as having jaundice, while the other 99.3% (1,258,889) did not have jaundice. The inclusion in jaundice group was based on having diagnosis of jaundice, present on admission, and not based on any particular level of serum bilirubin. We were unable to determine the mean serum bilirubin level in the study group. The mean age of patients with pancreatitis with jaundice was 57.8 ± 17.6 and 53.6 ± 16.8 in the other group. The study group had a higher proportion of males (>50%) and were predominantly Caucasian. As compared to the patients with pancreatitis and jaundice, the comorbidities, including chronic pulmonary disease (10.5% vs. 16.5%, *p* < 0.0001), hypertension (49.1% vs. 53.1%, *p* < 0.0001), diabetes mellitus (20.8% vs. 27.1%, *p* < 0.0001), hypothyroidism (7.2% vs. 8.5%, *p* < 0.0001), liver disease (8.6% vs. 15.2%, *p* < 0.0001), renal failure (8.3% vs. 19.7%, *p* < 0.0001), depression (7.1% vs. 13.8%, *p* < 0.0001), and alcohol abuse (16.9% vs. 23.7%, *p* < 0.0001), were higher in the group without jaundice. Other comorbidities like metastatic cancer (3.8% vs. 1.5%, *p* < 0.0001) and evidence of biliary stone (36.2% vs. 16.7%, *p* < 0.0001) were higher in the group presenting with jaundice. Patients without jaundice also had a higher proportion of prior tobacco use (32.7% vs. 37.9%, *p* < 0.0001). Patients with jaundice had a higher proportion of liver cancer (1.9% vs. 0.4%, *p* < 0.0001), PA (4.9% vs. 1.2%, *p* < 0.0001), and cholangiocarcinoma (1.5% vs. 0.1%, *p* < 0.0001) as compared to those without jaundice. Patient and hospital characteristics are outlined in Table 1. Confidence interval (CI) used for comparison and analysis was 99%.

### 3.2. Primary Outcomes: In-Hospital Mortality

When compared between the two groups, the in-hospital mortality was significantly higher in patients with pancreatitis with jaundice (4.3% vs. 2.1%, *p* < 0.0001) than those without. The study group had higher odds of in-hospital mortality (adjusted odds ratio [aOR]: 1.51, 99% CI 1.35–1.68, *p* < 0.0001) compared to pancreatitis patients without jaundice when adjusted for comorbidities (age, sex, ethnicity, Elixhauser Comorbidity Index, hospital location, hypertension, smoking history, diabetes, heart disease, chronic pulmonary disease, hypothyroidism, and renal and liver disease) using multivariate logistic regression analysis.

### 3.3. Secondary Outcomes

Patients with pancreatitis and jaundice had a higher incidence of sepsis (15.2% vs. 9.6%, *p* < 0.0001), septic shock (4.1% vs. 2.9%, *p* < 0.0001), ascites (6.5% vs. 3.1%, *p* < 0.0001), dehydration and electrolyte imbalance (47.6% vs. 43.8%, *p* < 0.0001), acute kidney injury (17.8% vs. 14.9%, *p* < 0.0001), and acute liver failure (1.6% vs. 1.1%, *p* < 0.0001) than those without jaundice. The total hospitalization cost was higher in patients with jaundice ($11,412 vs. $7893, *p* < 0.0001). There was no significant difference in median length of stay, the incidence of acute respiratory distress syndrome (ARDS), and ventilator-dependent respiratory failure (VDRF). The comparison of outcomes within the two groups is outlined in Table 2.

### 3.4. Subgroup Analysis

Out of a total number of patients admitted for pancreatitis with jaundice, a relatively small fraction (4.3%) underwent endoscopic retrograde cholangio-pancreatography (ERCP) during the hospital stay. When comparing the mortality of patients admitted with pancreatitis and jaundice who underwent ERCP with the patients who did not undergo ERCP, the adjusted mortality was significantly lower in the group that underwent ERCP during the admission. The indications for ERCP were not identified during the analysis. Odds of undergoing ERCP were calculated for both the study groups as demonstrated in Table 3. OR = 4.3% of 8855/0.9% of 1,258,889 = 4.45 (*p* < 0.0001).

## 4. Discussion

Acute pancreatitis is one of the most recognized causes of increased morbidity and mortality in hospitalized patients and is a leading cause of gastrointestinal-related admissions [1]. Some of the common causes of pancreatitis include gallstones, alcohol use, idiopathic, auto-immune, drug-induced, hypertriglyceridemia, and trauma. Gallstones are regarded as the major cause of pancreatitis and are responsible for roughly 70% of cases of acute pancreatitis [1]. Except for alcohol-induced and trauma, the etiology of pancreatitis is typically indistinguishable based on the clinical presentation.

Shaka et al. [14] reported that patients with biliary pancreatitis were reported to have higher inpatient mortality and morbidity as compared to those with pancreatitis due to hypertriglyceridemia. It was postulated that patients with pancreatitis due to hypertriglyceridemia have possibly better inpatient outcomes including mortality as compared to biliary pancreatitis. The same database was used to collect data from the period of 2016–2017 and conduct analysis like our study. Taking the results of this study into consideration, patients with gallstone pancreatitis warranted close monitoring for the development of sepsis, shock, NSTEMI, and transfusion dependence, especially after invasive procedures. In their study, they looked at different etiologies of pancreatitis and compared mortality considering different etiologies of the pancreatitis. Our study looked at all causes of pancreatitis collectively, unlike the study by Shaka et al. Their study did not comment on whether patients with biliary pancreatitis had jaundice on presentation, unlike our study. Their study also did not look at any difference in mortality considering the presence or absence of jaundice as a presenting symptom. Our observational study also showed that patients presenting with pancreatitis with jaundice tend to be of older age (57.8 ± 17.6 years vs. 53.6 ± 16.8 years) as compared to those without jaundice. In their study, they also mentioned that the length of stay was significantly higher in patients with biliary pancreatitis, 5.1 days versus 4.1 days. As per our study, there was no significant difference in the length of stay between the two groups.

McCollum and Jordan reported in a series 7 patients who had jaundice and pancreatitis in the absence of choledocholithiasis. In this situation, it was postulated that jaundice is likely the result of the hepatocellular injury [17]. The hepatocellular injury could likely be contributing to worse the outcomes in terms of mortality and morbidity as demonstrated in our study. Our study had a significantly higher sample size to the one by McCollum and Jordan. Unlike our study, their study did not mention about the association of worse outcomes with jaundice on presentation.

The role of ERCP in patients with pancreatitis is mostly reserved for those with evidence of choledocholithiasis, concomitant cholangitis, CBD dilation greater than 6 mm, and bilirubin level more than 4 mg/dl [18]. The study group of patients with pancreatitis with jaundice was sub-analysed into those who underwent ERCP during the admission as compared to those who did not undergo ERCP. The age-adjusted mortality showed mortality benefit in patients undergoing ERCP (2.3% vs. 4.3%, *p* < 0001). The results were consistent with the current standard of care of biliary pancreatitis with ongoing obstruction [18]. In our study, out of a total of 36% patients with jaundice and 16.7% patients without jaundice, 4.3% and 0.9% patients underwent ERCP, respectively, in both groups. When looked at numbers, seemingly higher number of patients without jaundice underwent ERCP (0.9% of 1,258,889 = 11,330) as compared to the patients with jaundice (4.3% of 8855 = 381). This is likely explained because of vast difference in the sample size of the two groups. When odds of undergoing ERCP are compared for the two groups, the patients with pancreatitis and jaundice had 4.45 times the odds of undergoing ERCP as compared to the patients without jaundice. The possible explanation for such small percentage of patients undergoing inpatient ERCP is likely due to the finding of sludge or small calculi that likely spontaneously migrated out of the bile duct. In our study, the patients who might have undergone outpatient ERCP like patients with PA were not accounted for. Our findings are consistent with current literature that urgent intervention is recommended in patients with cholangitis or active obstruction.

The cost associated with in-hospital stay was significantly higher in patients with pancreatitis with jaundice than those without jaundice ($11,412 vs. $7893, *p* < 0.0001). There was no significant difference in the median length of stay between the two groups. The information seems counter intuitive given that more patients with pancreatitis required procedures and had worse possible outcomes in the setting of a similar length of stay. The higher cost could be attributed to the higher incidence of complications, such as sepsis and septic shock, as well as higher requirements of procedures like ERCP and cholecystectomy.

Large-scale studies that have compared outcomes of pancreatitis with and without jaundice have been infrequent. There is a widespread use of prognostic markers for pancreatitis. Glasgow Pancreatitis score assesses the severity of pancreatitis within 48 hours from admission and includes pointers like age, serum albumin, arterial pO_2_, serum calcium, blood glucose, serum LDH, serum urea nitrogen (BUN), and WBC count [19]. BISAP score utilizes BUN, presence of encephalopathy, sepsis, and effusion, and age above 60 years as risk factors for worse outcomes [20]. Other similar prognostic scores are Ranson's criteria and APACHE II score. None of these prognostic factors include the level of bilirubin or jaundice as a prognostic factor for predicting worse outcomes. Our study did demonstrate that patients who had concomitant jaundice with pancreatitis had worse outcomes in terms of in-hospital mortality, sepsis, septic shock, dehydration, acute kidney injury, ascites, and acute liver failure. Table 2 shows the comparison of outcomes between the two groups.

Pancreatitis can be a presenting feature in PA, and Dzeletovic et al. deduced that it might lead to early diagnosis of PA. Their study demonstrated that a substantial proportion of PA patients have a history of pancreatitis and that PA patients with a history of pancreatitis are more likely to present at an earlier stage, have surgical resection, and therefore have improved survival. Their study was a retrospective study, like ours, which looked at cases of PA that were identified through review of the prospective Mayo Clinic Biospecimen Resource for Pancreas Research from January 1992 through September 2011. In their study, jaundice as a presenting symptom was not discussed [21]. In addition, whether presence of PA was associated with patients presenting with jaundice was not investigated. With the bigger sample size, our study showed a higher presence of PA in patients presenting with pancreatitis and concomitant jaundice as compared to those without jaundice (4.9% vs. 1.2%, *p* < 0.0001).

The limitations of the study are as follows. The analysis is limited by utilizing the discharge diagnoses and the billing codes of the NIS database that is subject to inaccuracy in documentations and missing codes. We are unable to determine the mean level of bilirubin in either of the study groups, and distribution in either of the groups was based on having ‘Jaundice' as one of the diagnosis on admission. There were significant differences in patient-level variables, hospital characteristics, and comorbidities between the two groups at baseline including higher incidence of pancreatic, liver, and biliary cancers, which could contribute to worse in-hospital mortality. We aimed to limit the differences at baseline running a multi-variate analysis, but this would not account for all possible confounders. In subgroup analysis, for the patients undergoing ERCP, the indication for performing ERCP was not investigated.

The main strengths of the study include large sample size and varied representation of the general population [15, 16]. Despite the limitations, our scientific questioning, large sample size, and analysis technique help contribute new information to a previously understudied topic of outcomes of pancreatitis with and without jaundice.

## 5. Conclusions

To our knowledge, this is the largest study to date looking at outcomes of patients with pancreatitis with and without jaundice. The patients admitted with pancreatitis with jaundice have significantly higher in-hospital mortality and morbidity, and higher healthcare expenditure as compared to those without jaundice. ERCP is the standard of care in patients with biliary pancreatitis with ongoing biliary obstruction. The results of our study show similar results that do ERCP during the same admission for patients with biliary pancreatitis, assuming there was an ongoing obstruction. Through this study, we hope to increase clinician suspicion for identifying jaundice as a negative prognostic marker in patients with pancreatitis and early identification of potential complications. We also look to encourage more large-scale prospective studies in identifying jaundice or elevated bilirubin as a marker of worse outcomes and potentially including it in prognostic scores. Further studies are recommended to identify reversible risk factors for jaundice in patients with pancreatitis to guide quality measures that would ultimately improve outcomes and reduce the cost of hospitalization.

## Figures and Tables

**Table 1:** Demographic characteristics of the two groups.

Characteristics	Pancreatitis with jaundice	Pancreatitis without jaundice	*p*-value for CI 99%
*N* = 1,267,744	*N* = 8855 (0.7%)	*N* = 1,258,889 (99.3%)	
**Age**			<0.0001
Mean years (mean ± SD)	57.8 ± 17.6	53.6 ± 16.8	
**Gender**			<0.0001
Male	59.1%	52.3%	
Female	40.9%	47.7%	
∗Missing—475			
**Age groups (years)**			<0.0001
18–44	23.7%	30.6%	
45–64	38.2%	42.9%	
65–84	32%	22.3%	
≥85	6.1%	4.1%	
**Race**			<0.0001
Caucasians	67.4%	61.9%	
African Americans	9.6%	16.8%	
Others	23%	21.3%	
∗Missing—10			
**Insurance type**			<0.0001
Medicare	40.9%	36.3%	
Medicaid	17.2%	24.3%	
Private	31.8%	27.9%	
Other	9.9%	11.4%	
∗Missing—75			
**Elixhauser comorbidities**			
Congestive heart failure	8.2%	7.4%	0.003
Peripheral vascular disease	3.3%	3.6%	0.25
Paralysis	1.1%	1.5%	0.001
Other neurological disorders	4.9%	6.8%	<0.0001
Chronic pulmonary disease	10.5%	16.5%	<0.0001
Hypertension	49.1%	53.1%	<0.0001
Diabetes mellitus	20.8%	27.1%	<0.0001
Hypothyroidism	7.2%	8.5%	<0.0001
Renal failure	8.3%	10.7%	<0.0001
Liver disease	8.6%	15.2%	<0.0001
Peptic ulcer disease chronic	2.6%	1.9%	<0.0001
Metastatic cancer	3.8%	1.5%	<0.0001
Solid tumor w/out metastasis	3.9%	1.7%	<0.0001
Coagulopathy	13.4%	8.9%	<0.0001
Weight loss	12.3%	9.8%	<0.0001
Fluid and electrolyte disorders	42.9%	39.9%	<0.0001
Chronic blood loss anemia	0.8%	0.7%	0.65
Deficiency anemias	17.7%	15.9%	<0.0001
Alcohol abuse	16.9%	23.7%	<0.0001
Drug abuse	3.3%	6.2%	<0.0001
Depression	7.1%	13.8%	<0.0001
**Liver cancer**	1.9%	0.4%	<0.0001
**Pancreatic cancer**	4.9%	1.2%	<0.0001
**Age of diagnosis (pancreatic cancer patients)**	62.7 ± 12.8	67.5 ± 10.5	
**Cholangiocarcinoma**	1.5%	0.1%	<0.0001
**Smoking**	32.7%	37.9%	<0.0001
**Antibiotic use**	0.2%	0.3%	0.05
**Biliary stone**	36.2%	16.7%	<0.0001
ERCP	4.3%0	0.9%	<0.0001
Endoscopic sphincterotomy	2.2%	0.2%	<0.0001
Percutaneous cholecystostomy	1.2%	0.3%	<0.0001
Cholecystectomy	19.5%	11.2%	<0.0001
**Admission type**			0.01
Emergent	93.7%	94.3%	
Elective	6.3%	5.7%	
∗Missing—2880			
**Hospital ownership/control**			<0.0001
Rural	7.8%	10%	
Urban nonteaching	26.3%	26.6%	
Urban teaching	65.9%	63.4%	

**Table 2:** Assessment of outcomes.

Outcomes	Pancreatitis with jaundice	Pancreatitis without jaundice	*p*-Value
In-hospital mortality
∗Missing—920	4.3%	2.1%	<0.0001
	1.51 (1.35–1.68)		<0.0001
Length of stay, days (mean, IQR)	4 (3–8)	4 (2–6)	0.0023
Total hospitalization cost, $ (median, IQR)	11,412 (6843–19,216)	7893 (4840–13,842)	<0.0001
**In-hospital complications**			
ARDS	0.4%	0.3%	0.08
Ventilator dependence respiratory failure	0.23%	0.29%	0.25
Dehydration and electrolyte disorders	47.6%	43.8%	<0.0001
Septicemia	15.2%	9.6%	<0.0001
Septic shock	4.1%	2.9%	<0.0001
Prolonged ileus	0.3%	0.2%	0.38
Ascites	6.5%	3.1%	<0.0001
Hypoalbuminemia	2.5%	1.4%	<0.0001
AKI	17.8%	14.9%	<0.0001
Peritonitis	1.8%	1.4%	0.0008
Perforation of intestine	0.06%	0.2%	0.006
Toxic megacolon	0.11%	0.17%	0.17
Acute liver failure	1.6%	1.1%	<0.0001
**Disposition**			<0.0001
Discharge to home	68.9%	74.9%	
Transfer other: Includes skilled	10.1%	8.6%	
Nursing facility (SNF), intermediate			
Care facility (ICF) and another type of facility			
Home health care	8.5%	8.1%	
Against medical advice (AMA)	1.6%	3.4%	
∗Missing—920			

Adjusted for age, race, gender, Elixhauser comorbidities, primary payer, and hospital teaching status.

**Table 3:** Outcome comparison between those patients who underwent ERCP versus those who did not.

	Patients with pancreatitis and jaundice who underwent ERCP (*n* = 381)	Patients with pancreatitis and jaundice who did not undergo ERCP (*n* = 8474)	*N* = 8855
In-hospital mortality
∗Missing—107	2.3%	4.3%	*p* < 0.0001
Length of stay (median, days)	4	4	*p* = 0.0023

## Data Availability

National Inpatient Sample (NIS) database.
